# Identifying monoclonal gammopathy of undetermined significance from electronic health records

**DOI:** 10.1002/cnr2.1755

**Published:** 2022-12-04

**Authors:** Hilary C. Tanenbaum, Brenda M. Birmann, Kimberly A. Bertrand, Lauren R. Teras, Amrita Y. Krishnan, Hoda Pourhassan, Scott Goldsmith, Kimberly Cannavale, Sophia S. Wang, Chun R. Chao

**Affiliations:** ^1^ Department of Research & Evaluation Kaiser Permanente Southern California Pasadena California USA; ^2^ Scientific Research & Development Embark Veterinary Ithaca New York USA; ^3^ Channing Division of Network Medicine, Department of Medicine Brigham and Women's Hospital and Harvard Medical School Boston Massachusetts USA; ^4^ Slone Epidemiology Center Boston University Boston Massachusetts USA; ^5^ Intramural Research Department American Cancer Society Atlanta Georgia USA; ^6^ City of Hope Medical Center California USA

**Keywords:** case identification, diagnosis code, electronic health records, MGUS, monoclonal gammopathy of undetermined significance

## Abstract

**Background:**

Monoclonal gammopathy of undetermined significance (MGUS) precedes multiple myeloma (MM). Use of electronic health records may facilitate large‐scale epidemiologic research to elucidate risk factors for the progression of MGUS to MM or other lymphoid malignancies.

**Aims:**

We evaluated the accuracy of an electronic health records‐based approach for identifying clinically diagnosed MGUS cases for inclusion in studies of patient outcomes/ progression risk.

**Methods and Results:**

Data were retrieved from Kaiser Permanente Southern California's comprehensive electronic health records, which contain documentation of all outpatient and inpatient visits, laboratory tests, diagnosis codes and a cancer registry. We ascertained potential MGUS cases diagnosed between 2008 and 2014 using the presence of an MGUS ICD‐9 diagnosis code (273.1). We initially excluded those diagnosed with MM within 6 months after MGUS diagnosis, then subsequently those with any lymphoid malignancy diagnosis from 2007 to 2014. We reviewed medical charts for 100 randomly selected potential cases for evidence of a physician diagnosis of MGUS, which served as our gold standard for case confirmation. To assess sensitivity, we also investigated the presence of the ICD‐9 code in the records of 40 randomly selected and chart review‐confirmed MGUS cases among patients with a laboratory report of elevated circulating monoclonal (M‐) protein (a key test for MGUS diagnosis) and no subsequent lymphoid malignancy (as described above).

The positive predictive value (PPV) for the ICD‐9 code was 98%. All MGUS cases confirmed by chart review also had confirmatory laboratory test results. Of the confirmed cases first identified via M‐protein test results, 88% also had the ICD‐9 diagnosis code.

**Conclusion:**

The diagnosis code‐based approach has excellent PPV and likely high sensitivity for detecting clinically diagnosed MGUS. The generalizability of this approach outside an integrated healthcare system warrants further evaluation.

## 1 INTRODUCTION

Monoclonal gammopathy of undetermined significance (MGUS) is a largely asymptomatic obligate precursor to multiple myeloma (MM) and other lymphoproliferative disorders. Clinically, MGUS features moderately elevated serum monoclonal protein (M‐protein, <3 g/dL) and the absence of evidence of end organ damage (i.e., hypercalcemia, renal failure, anemia and bone lesions) or amyloidosis.^1^, ^2^ It is distinguished from smoldering MM, an intermediate stage between MGUS and MM by the M‐protein level (M‐protein ≥3 g/dl for smoldering MM) and the abundance of plasma cells in the bone marrow.^1^ The prevalence of MGUS among adults aged 50 years or older in the United States is estimated at approximately 3%, with 1% of all MGUS cases advancing to MM per year.^2^, ^3^

Overall, factors relating to the progression of MGUS to MM and other lymphoproliferative disorders remains largely unknown. Additionally, MGUS patients have demonstrated higher morbidity and mortality from bacterial and viral infections, peripheral neuropathy, thrombosis and other chronic diseases.^4^, ^5^, ^6^ Further epidemiologic investigation of MGUS would facilitate advances in knowledge of MGUS disease progression and the development of strategies to prevent several malignant and non‐malignant outcomes.

While population‐based screening may be considered the gold standard for observational research, this approach requires the availability of archived biospecimens. For MGUS, the diagnosis is not clinically actionable at present,^1^ and thus widespread clinical screening to detect MGUS is not justifiable. Moreover, laboratory assays are expensive and therefore may not be feasible to use for broad‐scale screening to detect MGUS. Alternatively, manual medical chart review could be conducted to confirm MGUS diagnoses, but this approach is time‐ and labor‐intensive and would be prohibitively expensive for use in large epidemiological studies. Additionally, issues such as variation in reviewers' attention to detail can introduce ascertainment errors.^7^ More recently, electronic algorithms have been developed to identify MGUS cases from electronic health records using diagnosis and utilization codes (e.g., for oncologist visit[s] and relevant lab tests without incorporating lab results). Studies of such algorithms have reported positive predictive values (PPV) between 76% and 88%.^8^, ^9^

To build on these efforts to facilitate large scale epidemiologic research of MGUS using electronic health records—in particular, studies of factors associated with risk of progression to malignancy or other outcomes—we evaluated the performance of an electronically searchable diagnosis code‐based algorithm to identify patients with clinically diagnosed MGUS using electronic health records from a large integrated health care delivery system.

## 2 METHODS

### 2.1 Study setting

This study was conducted at Kaiser Permanente Southern California, a large integrated healthcare system with over 4.6 million members. Data were retrieved from the system's comprehensive electronic health records, which contain chart notes from all medical encounters (including outpatient visits, emergency room visits, and hospitalizations), laboratory testing data and diagnosis codes, and Kaiser Permanente's Surveillance, Epidemiology and End Results‐affiliated cancer registry. The study was approved by Kaiser Permanente Southern California's Institutional Review Board, which also waived the requirement for informed consent.

### 2.2 MGUS case identification algorithm and eligibility criteria for chart review confirmation

The algorithm that we evaluated for identifying patients with clinically diagnosed MGUS had the following steps:

- We searched for patients with a first ICD‐9 diagnosis code of 273.1 between 2008–2014 (e.g., the “index” ICD‐9 code).
- We excluded those with a MM diagnosis within 6 months following the record of the index ICD‐9 code.^10^
- Of the potential MGUS cases identified by steps (i) and (ii), we further restricted to those with at least 1 year of continuous health plan membership prior to the date of the index ICD‐9 code for the manual chart review confirmation (so that sufficient medical records would be available to confirm the MGUS diagnosis).
- We then randomly sampled 100 individuals from the remaining sample of eligible putative MGUS cases for chart review.

The initial chart reviews revealed that some recorded electronic ICD‐9 codes for MGUS corresponded to a work‐up that led to diagnoses of other lymphoid malignancies (since M‐protein may also be used to monitor disease status in patients with other lymphoid malignancies^10^, ^11^). We thus subsequently revised the case‐identification algorithm outlined above to further restrict the sample of potential cases to those without evidence of other lymphoid malignancies from 2007 to 2014 and applied the same revision to the randomly selected subsample.

When developing the case‐identification algorithm, we had considered developing a second algorithm based on records reporting serum M‐protein and immunofixation test results indicative of MGUS. We found that while serum M‐protein results can be queried as a discrete data field in Kaiser Permanente Southern California's electronic health records, they are sometimes not quantifiable, hindering their interpretation to determine the presence or absence of MGUS or a more advanced condition.^2^ Further, immunofixation results exist only as free text and thus cannot be readily queried without language processing tools that were not available to the project. Given these limitations, we could not develop a comprehensive case‐identification algorithm based on laboratory results. Nonetheless, we used the initial M‐protein‐based efforts to identify a separate sample of plan members with clinician‐diagnosed MGUS in whom we could assess the sensitivity of the ICD‐9 diagnosis‐code based approach, as described below.

### 2.3 Chart review confirmation for clinically diagnosed MGUS among individuals with an ICD‐9 diagnosis code for MGUS

We manually reviewed medical chart notes within (±) 6 months of the first recorded ICD‐9 code (273.1) for documentation of a physician diagnosis to confirm clinically diagnosed MGUS for the randomly selected putative cases. For any unconfirmed cases, we further conducted review of the entire medical history to understand potential reasons for the inaccuracies. All reviews and confirmation dispositions were verified by a second chart reviewer. Because our purpose was to validate the diagnosis code‐based algorithm for identifying patients with clinically diagnosed MGUS (rather than to ascertain all diagnosed and undiagnosed MGUS in the Kaiser Permanente Southern California population or to determine the accuracy of the physician diagnosis against standard diagnostic criteria for MGUS^2^), a physician diagnosis of MGUS in the chart notes was considered the gold standard for confirmation.

During chart review, we collected information on relevant test results, including serum or urine M‐protein, immunofixation and free light chain tests when available, and on the presence of clinical signs of end organ damage that contribute to a diagnosis of full‐blown MM, such as hypercalcemia, renal failure, anemia and bone lesions. The latter information was not always documented, and when present, the underlying conditions leading to the associated form of end organ damage were often not specified (e.g., renal failure could be due to long‐term diabetes rather than to MM or other malignancy). These challenges supported our decision to rely on evidence of a physician diagnosis as the gold standard for confirming clinically diagnosed MGUS in plan members with the corresponding ICD‐9 code rather than relying on reported clinical symptoms. We initially sought to determine the timing of the physician diagnosis. However, as the chart notes often had insufficient documentation of this timing, it was rarely possible to distinguish patients with newly diagnosed MGUS from those with a history of MGUS that predated our study period.

To address potential misclassification between MGUS and smoldering MM, we specifically searched the chart notes to capture potential smoldering MM diagnoses for those without a quantifiable M‐protein value (e.g., whose smoldering MM would have remained undetected by a review of M‐protein test results). We also searched for and conducted chart review to confirm an electronic ICD‐9 code for MM diagnosis (203.0) within 2 years after the index date among all confirmed MGUS cases, as misclassified smoldering MM cases would have a higher probability than true MGUS cases of progressing that quickly after the MGUS diagnosis.^1^, ^12^

### 2.4 Estimation of the sensitivity of the ICD‐9‐based MGUS case identification algorithm

To estimate the sensitivity of the electronic ICD‐9 code‐based case‐identification algorithm, we used the same process described above (for the subsample of putative cases first identified with the ICD‐9 diagnosis code‐based approach) to perform chart review of 54 randomly selected putative MGUS patients first identified based on a first serum M‐protein test result between 0 and 3 g/dL between 2008 and 2014 (“index” M‐protein test), with at least 1 year of health plan membership prior to the index M‐protein test result and with no subsequent diagnosis of MM or other lymphoid malignancy (assessed as described for the ICD‐9‐based algorithm above). We utilized the subset of these 54 health plan members who were confirmed by medical chart review to have clinically diagnosed MGUS as a separate patient sample in whom to estimate the sensitivity of the ICD‐9 code‐based algorithm. Specifically, we searched those patients' electronic records for an ICD‐9 diagnosis code for MGUS (273.1) before or within 1 year after the index M‐protein test. A more comprehensive evaluation of algorithm sensitivity was beyond the scope of this project.

### 2.5 Hematologist adjudication to explore the accuracy of the clinician diagnosis of MGUS

As an exploratory exercise to assess the accuracy of the MGUS diagnosis made by physicians in the chart notes in comparison to current MGUS diagnostic criteria,^2^ two hematologists (co‐authors Hoda Pourhassan and Scott Goldsmith) independently adjudicated 10 randomly selected chart review confirmed MGUS cases. This chart review process was a separate exercise from the steps described above to confirm the index ICD‐9 diagnosis code for MGUS via medical chart review. The two hematologists reviewed relevant clinical information available within 6 months of the index MGUS diagnosis code and provided an assessment of their certainty of the presence of MGUS by designating the putative MGUS case as “definite, probable, possible, no evidence of MGUS, or unable to determine.” They also provided notes articulating the rationales for their assessments. Discrepancy between the two hematologists was resolved by discussion. A priori, we considered the cases adjudicated as “definite” and “probable” MGUS as confirmed cases and those with “possible,” “no evidence of MGUS,” or “unable to determine” as unconfirmed cases according to current diagnostic criteria.

### 2.6 Statistical analysis

The distributions of demographic characteristics (age, sex, race/ethnicity) and the Charlson comorbidity index were obtained for the subsample of 100 ICD‐9 algorithm‐identified potential cases randomly selected for chart review. We also utilized the chart review findings to calculate the PPV for this subsample to inform the probability that an MGUS patient identified by the electronic record ICD‐9 code‐based algorithm truly had a physician diagnosis of MGUS (the gold standard for case confirmation for this project). Specifically, among the subsample of ICD‐9 algorithm‐identified putative MGUS cases subjected to chart review, the PPV was calculated as:

$$\mathrm{PPV}=\begin{array}{l} \left(\#\text{of putative MGUS cases confirmed}\;\mathrm{by}\;\text{chart review to}\;\mathrm{be}\;\text{clinically diagnosed}\right)\\ ÷\left(\#\text{of}\;\mathrm{all}\;\text{potential MGUS cases subjected to chart review}\right). \end{array}$$

We calculated a second PPV among the further restricted subsample after applying the exclusion criterion based on a diagnosis of any lymphoid malignancy during the study period using the same formula as above. For that second PPV calculation, the additional exclusion criterion was applied to both the denominator and the numerator of the calculation. In the separate subset of putative MGUS cases first identified by the M‐protein test result‐based alternative algorithm (described above) and subsequently confirmed by medical chart review to have a clinician diagnosis of MGUS, we estimated the sensitivity of the ICD‐9 code‐based algorithm as:

$$\text{Sensitivity}=\begin{array}{l} (\#\text{of putative MGUS cases confirmed}\;\mathrm{by}\;\text{chart review to}\;\mathrm{be}\;\text{clinically diagnosed and}\;\mathrm{who}\;\mathrm{had}\;\text{an electronically recorded}\;\mathrm{ICD}\\ -\;9\;\text{code of}\;273.1\;\text{before or within}\;1\;\text{year after the}\;M\\ -\;\text{protein test})÷(\#\text{of}\;\mathrm{all}\;\text{putative MGUS cases initially identified}\;\mathrm{by}\;a\;M\\ -\;\text{protein test and confirmed}\;\mathrm{by}\;\text{chart review to}\;\mathrm{be}\;\text{clinically diagnosed}). \end{array}$$

## 3 RESULTS

### 3.1 Putative MGUS patients identified by the ICD‐9 code‐based case‐identification algorithm

**FIGURE 1:**
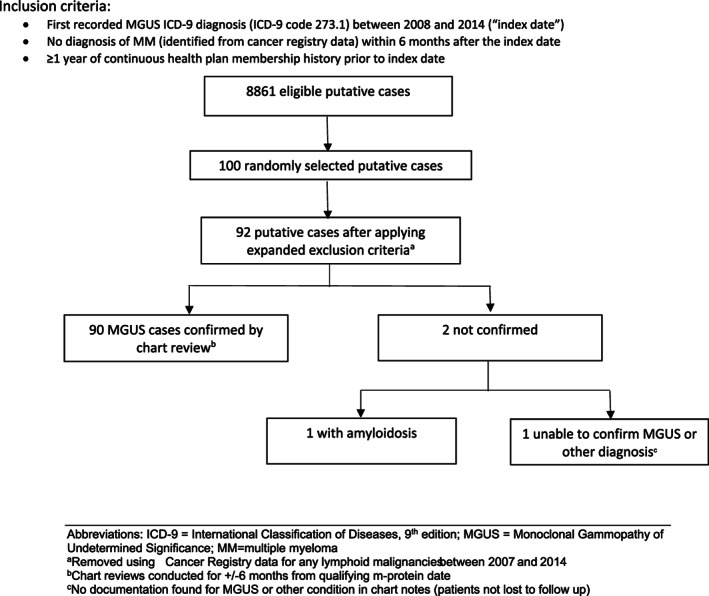
Flowchart for ICD‐9 code‐based case identification and chart review confirmation

A total of 8861 potential MGUS cases who met the eligibility criteria were identified using the case‐identification algorithm. Of these, we randomly selected 100 cases for chart review confirmation. Of these 100 potential cases, 58 were male, and 42 were of non‐Hispanic white race/ethnicity (Table 1). The mean age as of the index ICD‐9 diagnostic record was 72 years (standard deviation [SD]: 10.4). The mean Charlson comorbidity index score was 2.7. Prior to applying the additional exclusion criterion related to a diagnosis of other lymphoid malignancies during the study period, 90 of the 100 randomly selected putative MGUS patients had evidence of a physician diagnosis of MGUS in the medical chart, corresponding to a PPV of 90% for the initial ICD‐9 code algorithm (Table 2). After we applied the additional exclusion criterion 92 putative cases remained. Among those, 90 (PPV = 97.8%, Table 2) were still confirmed in chart notes, and 2 (2.2%) were not confirmed (1 had an amyloidosis diagnosis and 1 had no discernible relevant diagnosis, Figure 1).

**TABLE 1:** Demographic and clinical characteristics of the Kaiser Permanente Southern California plan members initially identified as putative MGUS patients by an ICD‐9 code‐based algorithm and subsequently randomly selected for confirmation of the MGUS diagnosis by medical chart review

	Chart review confirmation subsample (*N*=100)^a^
Age, years, mean (SD)	72.0 (10.4)
Male sex	58 (58.0%)
Race/ethnicity	
Non‐Hispanic white	42 (42.0%)
Non‐Hispanic black	24 (24.0%)
Hispanic	22 (22.0%)
Asian/Pacific islander	8 (8.0%)
Other/unknown	4 (4.0%)
Charlson comorbidity index, mean (SD)	2.7 (2.0)
Length of health plan membership, years, mean (SD)	24.1(14.6)

Abbreviations: ICD‐9, International Classification of Diseases, 9^th^ edition; MGUS, Monoclonal Gammopathy of Undetermined Significance; SD, Standard deviation.

^a^ These 100 individuals were randomly selected from all putative MGUS cases identified by the original algorithm, that is, as health plan members with a first (“index”) ICD‐9 code in the electronic record between 2008 and 2014, no subsequent diagnosis of MM (within 6 months after the index ICD‐9 code date), and at least 1 year of continuous plan membership prior to the index ICD‐9 code.

**TABLE 2:** Positive predictive value of the electronic record ICD‐9 code (273.1)‐based MGUS case identification algorithm in the Kaiser Permanente Southern California electronic health records system

Chart review result	Full subsample (*N*)^a^	Revised subsample (*N*)^b^
Confirmed^c^	90	90
Non‐confirmed	10	2
Total	100	92
Algorithm PPV^d^	90/100 = 90%	90/92 = 97.8%

Abbreviations: ICD‐9, International classification of diseases, 9^th^ Revision; MGUS, monoclonal gammopathy of undetermined significance; MM, multiple myeloma; PPV, positive predictive value.

^a^ This algorithm identified putative MGUS cases as health plan members with a first (“index”) ICD‐9 code in the electronic record between 2008‐2014, no subsequent diagnosis of MM (within 6 months after the index ICD‐9 code date), and at least 1 year of continuous plan membership prior to the index ICD‐9 code.

^b^ The revised algorithm further excluded putative MGUS cases with a diagnosis of any other lymphoid malignancy during 2007–2014.

^c^ We defined confirmed cases as those with evidence in the medical chart of a physician diagnosis of MGUS.

^d^ The PPV suggests that ~98% of health plan members identified by the algorithm summarized above truly had a physician diagnosis of MGUS (our “gold standard” for defining a confirmed case of MGUS).

### 3.2 Chart review findings on the confirmed clinically diagnosed MGUS cases

When we evaluated serum M‐protein test results for the 90 confirmed cases identified by the ICD‐9 code algorithm, we found a serum M‐protein result of <3 g/dl for 48 cases (42.4%). The charts for the 42 cases with no M‐protein lab value contained comments indicating that the serum protein electrophoresis result was not quantifiable; however, other chart information indicated that these cases were, in fact, all confirmed by immunofixation and/or free light chain testing to be true MGUS cases.

When we investigated the potential misclassification of smoldering MM as MGUS among the confirmed cases with no quantifiable M‐protein in the chart, we did not find any mention of a smoldering MM diagnosis within the chart review period. Of the full set of 90 confirmed MGUS cases, 3 developed MM within 2 years after the index ICD‐9 code date.

### 3.3 Sensitivity estimation

In the analyses to estimate the sensitivity of the ICD‐9‐based algorithm, 40 of the 54 potential MGUS cases initially identified by an eligible M‐protein test results were confirmed to be true clinically diagnosed MGUS cases by manual chart review. Of those 40 confirmed clinically diagnosed MGUS cases, 35 had an ICD‐9 diagnosis code before or within 1 year after the index M‐protein test, corresponding to an estimated sensitivity of 87.5% (Table 3).

**TABLE 3:** Sensitivity of the electronic record ICD‐9 code (273.1)‐based case identification algorithm as estimated in a separate subset of Kaiser Permanente Southern California plan members with physician‐diagnosed MGUS

ICD‐9 code in the electronic record^a^	MGUS cases confirmed by chart review (*N*)^b^
Yes	35
No	5
Total	40
ICD‐9 code algorithm sensitivity^c^	35/40 = 87.5%

Abbreviations: ICD‐9, International classification of diseases, 9^th^ Revision; MGUS, monoclonal gammopathy of undetermined significance; MM, multiple myeloma; PPV, positive predictive value.

^a^ ICD‐9 code in the electronic record between 2008‐2014, no subsequent diagnosis of MM (within 6 months after the index ICD‐9 code date) or of any other lymphoid malignancy (during 2007–2014), and at least 1 year of continuous plan membership prior to the index ICD‐9 code.

^b^ This subset of MGUS patients comprised health plan members with at least 1 year of continuous plan membership prior to an initial finding of an electronic record with a circulating monoclonal protein test result consistent with an MGUS diagnosis (0 to 3 g/dl) between 2007 and 2014 and with no subsequent diagnoses of MM (within 6 months of the qualifying lab result) or of any other lymphoid malignancy (during the study period) and for whom subsequent medical chart review yielded evidence of a physician diagnosis of MGUS.

^c^ This estimate suggests that ~88% of health plan members with a physician diagnosis of MGUS in the medical chart will also have an electronic record with an MGUS‐specific ICD‐9 code (273.1).

### 3.4 Hematologist adjudication findings

The adjudication of the 10 hematologist‐reviewed cases concluded 5 cases as “probable.” The remaining 5 were designated as “possible” due to the lack of complete information on the presence or absence of end organ damage (e.g., hypercalcemia, renal failure, anemia, and bone lesions) and/or the lack of findings from a bone marrow study. The unavailability of those details in the medical charts prevented the hematologists from ruling out a more advanced diagnosis than MGUS, for example, smoldering MM or MM, for the “possible” cases. (The hematologists did not note any findings that refuted the presence of either MGUS, smoldering MM or MM in any of the 10 charts they reviewed.)

## 4 DISCUSSION

We confirmed an algorithmic approach that can be used to efficiently and accurately identify clinically diagnosed prevalent MGUS cases for population‐based research to study outcomes of MGUS and factors associated with progression to malignancy. Our results suggest that the diagnosis code‐based algorithm has excellent PPV and likely also satisfactory sensitivity for ascertaining individuals with clinically diagnosed MGUS.

Our findings are aligned with results of a Danish study that used ICD diagnosis codes to identify patients with MGUS who were subsequently confirmed with chart reviews.^9^ That study reported an initial PPV of 82.3% but subsequently applied additional exclusion criteria to eliminate cases diagnosed with malignant monoclonal gammopathy prior to or within 1 year of the MGUS diagnosis. This change resulted in a PPV improvement of approximately 4 percentage points. Similarly, when we expanded our exclusion criteria to include other lymphoid malignancies, the PPV of our diagnosis code approach increased from 90% to 97.8%.

More recently, an algorithm was developed to identify MGUS cases using electronic health records from a large, community‐based healthcare group.^8^, ^13^ The criteria required at least two MGUS diagnosis codes entered on different dates in a 12‐month period, as well as at least one serum protein electrophoresis or immunofixation test (regardless of the results) and an oncologist office visit within 90 days of the MGUS diagnosis. After excluding putative patients diagnosed with MM ±3 months after the first MGUS diagnosis code, this approach achieved a PPV of 75%. It is possible that amending the exclusion criteria by extending the window of time and list of relevant diagnoses (e.g., to other lymphoproliferative disorders) may improve the PPV of this approach as well.

The hematologist adjudication findings suggested that MGUS diagnoses made in the community‐based practice may not always involve a complete symptom work‐up and/or bone marrow study within an applicable time window (i.e., a ±6‐month chart review window). Furthermore, other underlying conditions can contribute to the reporting of certain forms of MM‐defining end organ damage (e.g., poorly controlled diabetes for renal failure), yet the potential causes for the end organ damage are not always discussed in the chart. These practice patterns are likely also present in other community‐based practice settings. This finding suggests potential misclassification between MGUS and smoldering MM in real‐world practice, although the apparently missing clinical workups may have been completed at a time outside of the chart review window utilized for this study. We attempted to assess the magnitude of this potential misclassification by searching for MM diagnoses within 2 years after the index date. Of the 90 confirmed MGUS cases, only 3 developed MM within 2 years. This observation suggests that the potential misclassification of smoldering MM as MGUS by our ICD‐9‐based algorithm is small in magnitude given that the progression rate to MM for smoldering MM is expected to be much higher, for example, 10% per year for the first 5 years after diagnosis, whereas that for MGUS is ~1% per year.^14^

There are several other limitations to consider when interpreting our results. First, this study was not designed to assess the specificity or negative predictive value of our algorithms because of the focus on the reliable identification of individuals with a physician diagnosis of MGUS for inclusion in studies of outcomes of MGUS. For that purpose, the PPV and sensitivity were the most relevant parameters to evaluate. Second, we could not distinguish newly made MGUS diagnoses from those previously known due to insufficient documentation about the timing of a first MGUS diagnosis in the chart notes. Of note, since MGUS is an asymptomatic condition, the exact timing of MGUS onset cannot be determined for any patient in usual clinical care. A strength of our approach is that it allows the identification of a large proportion (potentially the vast majority) of clinically diagnosed MGUS patients at any given point in time. As an additional limitation, we note that our study period ended prior to the transition from ICD‐9 to ICD‐10 code use, which occurred in 2015. Potential differences in relevant code(s) in the ICD‐10 system should be considered when applying the ICD‐based algorithm to more recent years, as should the incorporation (around 2014) of serum free light chain testing into standard clinical workups to diagnose MGUS.^2^ Validation of an updated version of the algorithm could be informative to ensure optimal sensitivity and PPV for studies that span beyond 2014, while the inclusion of cases from earlier time periods based on the present ICD‐9 code algorithm can help to maximize follow‐up time for the study of relevant outcomes. Finally, the generalizability of our findings to different settings with electronic health records (such as in health systems that are not integrated) needs further confirmation.

Although we designed this study to validate ascertainment of clinically diagnosed MGUS, it should be noted that undiagnosed MGUS is prevalent in older populations; indeed, approximately 80% of prevalent MGUS cases are unrecognized.^15^ Thus, identification of MGUS patients from clinical diagnoses will likely lead to under‐ascertainment of the true MGUS prevalence. As a result, this case‐identification approach should not be used to identify all incident MGUS for etiologic studies. Rather, this approach will be useful to study clinically diagnosed MGUS, particularly with regard to disease progression and factors associated with disease progression. It should also be noted that asymptomatic patients are not randomly selected to undergo the laboratory tests that comprise a MGUS work up/diagnosis. Further research is necessary to evaluate or document factors that drive laboratory testing among asymptomatic individuals and characterize the potential bias of this non‐random MGUS detection in epidemiologic studies that are not designed as screening studies. For example, it is likely that seemingly healthier individuals (i.e., those without any clinical indication for serum protein electrophoresis testing) will be systematically missed in observational studies. The impact of this potential bias on electronic health record‐based studies of MGUS will need to be considered based on the specific study objectives.

Despite some limitations, our findings may be useful for certain types of epidemiologic studies, such as to investigate risk factors for MGUS malignant progression or to improve clinical surveillance for more serious non‐malignant outcomes. Such studies could accelerate identification of prevention strategies for more severe MGUS outcomes that would be considered clinically actionable, including MM and lymphoproliferative disorders. Results from the latter could be particularly useful for justifying larger screening‐based studies to develop risk‐prediction models among individuals with MGUS.

## AUTHOR CONTRIBUTIONS

**Hilary C Tanenbaum:** Data curation (lead); formal analysis (lead); investigation (lead); project administration (equal); validation (equal); writing – original draft (equal). **Brenda M Birmann:** Conceptualization (equal); funding acquisition (equal); methodology (equal); writing – review and editing (lead). **Kimberly Bertrand:** Methodology (supporting); writing – review and editing (supporting). **Lauren Teras:** Methodology (supporting); writing – review and editing (supporting). **Amrita Y Krishnan:** Methodology (supporting); writing – review and editing (supporting). **Hoda Pourhassan:** Investigation (supporting); writing – review and editing (supporting). **Scott Goldsmith:** Investigation (equal); writing – review and editing (equal). **Kim Cannavale:** Investigation (supporting); project administration (equal); validation (equal); writing – review and editing (supporting). **Sophia S Wang:** Conceptualization (equal); funding acquisition (equal); methodology (supporting); writing – review and editing (supporting). **Chun Chao:** Conceptualization (equal); methodology (equal); supervision (lead); writing – original draft (equal).

## CONFLICT OF INTEREST

The authors have stated explicitly that there are no conflicts of interest in connection with this article.

## ETHICS STATEMENT

This study is approved by Kaiser Permanente Southern California IRB, Approval # 11139.

## Data Availability

Anonymized data that support the findings of this study may be made available from the corresponding author on reasonable request from qualified researchers with documented evidence of training for human subjects protections.
